# Human papillomavirus-associated small cell carcinoma/neuroendocrine carcinoma of the oropharynx: a report of two cases

**DOI:** 10.1186/s40064-016-3501-x

**Published:** 2016-10-22

**Authors:** Kiyoshi Misawa, Hideya Kawasaki, Rieko Matsuo, Kenichi Sugiyama, Daiki Mochizuki, Shiori Endo, Atushi Imai, Yuki Misawa, Takashi Yamatodani, Kunihiro Mizuta, Hiroyuki Mineta

**Affiliations:** 1 Department of Otolaryngology/Head and Neck Surgery, Hamamatsu University School of Medicine, 1-20-1 Handayama, Hamamatsu, Shizuoka 431-3192 Japan; 2 Department of Regenerative and Infectious Pathology, Hamamatsu University School of Medicine, Hamamatsu, Japan

## Abstract

**Introduction:**

Small cell carcinoma/neuroendocrine carcinoma (SCNEC) of the oropharynx is uncommon. Two cases of SCNEC in an 81-year-old woman and in a 54-year-old man are presented here.

**Case description:**

We have documented two cases of SCNEC arising in the oropharynx with evidence of high-risk human papillomavirus (HPV) infection. Histologically, both cases were classified as poorly differentiated SCNEC with high nuclear-to-cytoplasmic ratios and nuclear molding. Observations using a transmission electron microscope revealed membrane-bound neuroendocrine granules in some tumor cells. Both tumors expressed high levels of p16, a surrogate marker for high-risk HPV infection. HPV infection was confirmed in both cases using HPV polymerase chain reaction analysis; HPV subtype 16 was identified in one case and HPV subtype 18 in the other.

**Discussion and Evaluation:**

SCNEC of the oropharynx is a rare and novel HPV-associated disease with neuroendocrine granules and aggressive clinical behavior.

**Conclusions:**

Herein, we present two cases of SCNEC, focusing on its histologic features and treatment modalities. More studies are required to elucidate the pathophysiology of HPV-associated SCNEC in different organ systems.

**Electronic supplementary material:**

The online version of this article (doi:10.1186/s40064-016-3501-x) contains supplementary material, which is available to authorized users.

## Background

Primary malignant tumors of the oropharynx are usually squamous cell carcinomas (SqCCs). Over the past decade, human papillomavirus (HPV) infection has been recognized as a significant etiological factor for a subset of oropharyngeal SqCCs. Primary small cell carcinoma/neuroendocrine carcinoma (SCNEC) of the oropharynx is rare. The larynx is the most commonly involved site, followed by the nasal cavity, paranasal sinuses, salivary glands, and oral cavity (Renner 2007; Mineta et al. 2001). Approximately 75 cases of SCNEC in the primary nasal/paranasal cavities and 180 cases in the larynx have been reported (Sirsath et al. 2013). The prognosis of SCNEC in the nasal cavity and larynx is poor (Chai et al. 2014). Recently, SCNEC of the uterine cervix and anus have been shown to be associated with HPV infection, and aggressive behavior of HPV-positive SCNEC in the female genital tract has been documented (Mills 2002). Herein, we present the clinical courses of two patients with oropharyngeal HPV-associated SCNEC.

## Case 1

**Fig. 1:**
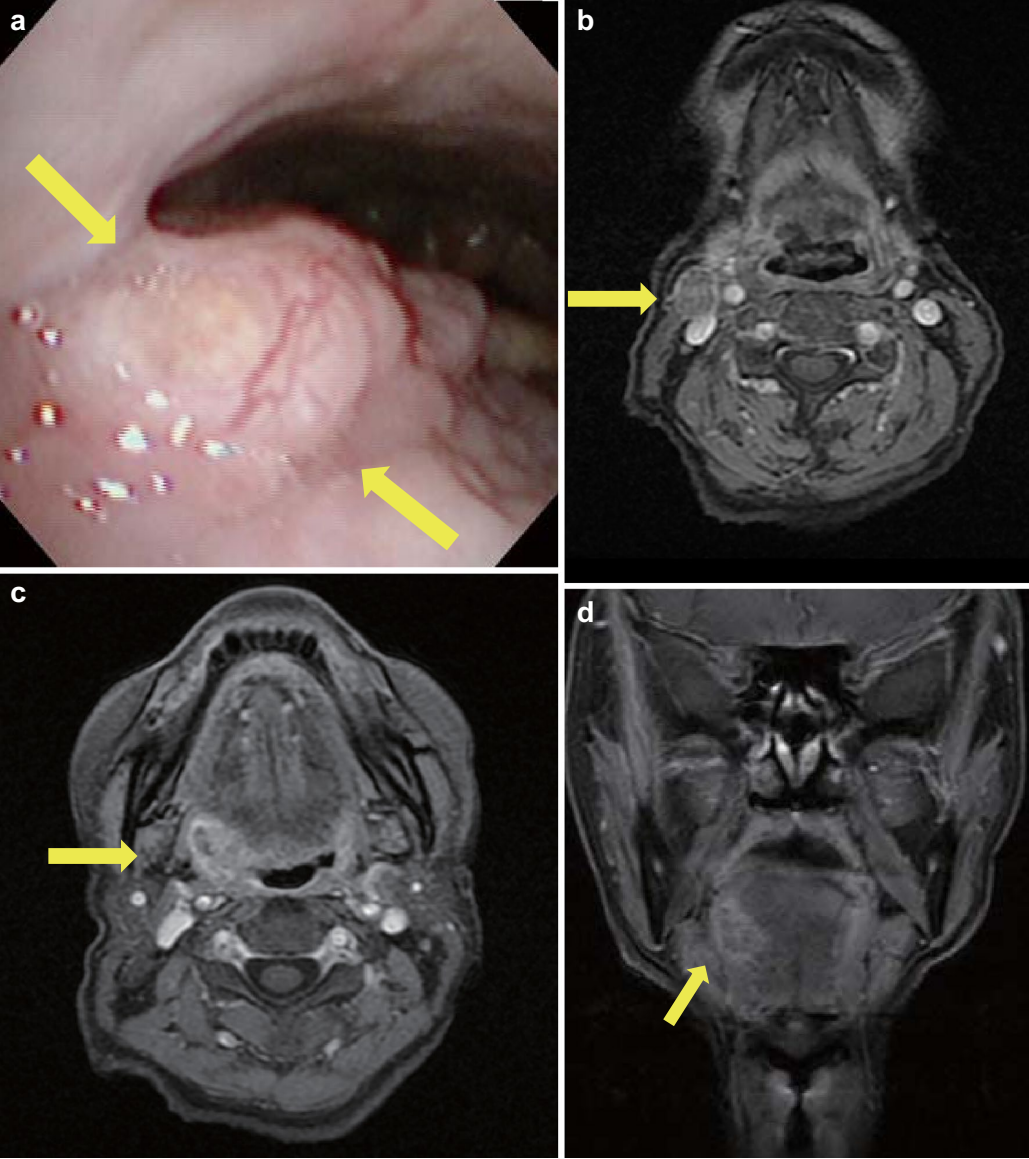
Case 1. **a** Oropharyngeal fiberscopic image. A tumor was observed in the right anterior wall of the oropharynx (*yellow arrow*). **b** Contrast-axial magnetic resonance imaging (MRI) scan of the neck before treatment. An enhanced metastatic tumor was observed in the right internal jugular node (*yellow arrow*). **c** Contrast-axial MRI scan of the neck before treatment. An enhanced primary tumor was observed in the right anterior wall of the oropharynx (*yellow arrow*). **d** Contrast-coronal MRI scan (*yellow arrow*)

An 81-year-old woman with no history of alcohol consumption or smoking presented with a 3-month history of experiencing a mass in her throat and right-sided neck swelling. On oropharyngoscopy, a tumor was identified in the right anterior wall of the oropharynx (Fig. 1a). Magnetic resonance imaging (MRI) of the neck revealed a 12 × 15-mm right swollen internal jugular node (Fig. 1b) and a 22 × 16 × 24-mm heterogeneously enhanced tumor that extended through the right anterior wall of the oropharynx (Fig. 1c, d). Fine needle aspiration cytology of a clinically palpable right level II lymph node showed features consistent with SqCC. No SCNEC component was identified in the limited biopsy sample obtained from the tumor. The tumor was positive for AE1/AE3 and negative for CD3, CD56, CD79a, synaptophysin, and chromogranin A expression. Subsequently, a pathologist analyzed the biopsy sample and diagnosed the lesion as SqCC. A partial pharyngectomy with right neck dissection was performed, and the malignancy was diagnosed as SCNEC. Histologic examination revealed small, round to oval tumor cells arranged in cords or nests, containing hyperchromatic nuclei and mitotic figures; the tumor was positive for synaptophysin and CD56 and negative for chromogranin A expression (Table 1). The patient was diagnosed with T2N1M0 oropharyngeal SCNEC, according to the 2009 Union for International Cancer Control staging system. After surgery, the patient refused radiotherapy and chemotherapy. Patient details are summarized in Table 1.

**Table 1:** Summary of the demographic details, immunohistochemistry profiles, and results of HPV tests for Cases 1 and 2

	Case 1	Case 2
Year of diagnosis	2010	1995
Age at diagnosis (years)	81	54
Sex	Female	Male
Primary site	Base of tongue	Left tonsil
TN stage	T2N1	T2N2b
Treatment	Surgery	Irradiation (70 Gy) + chemotherapy (cisplatin + etoposide)
Synaptophysin	Positive	Negative
Chromogranin A	Negative	Positive
CD56	Positive	Positive
TEM	Granules	Granules
p16	Positive	Positive
HPV test	Type16	Type18
Follow-up interval (month)	22	10
Outcome	Dead of disease	Dead of disease

## Case 2

**Fig. 2:**
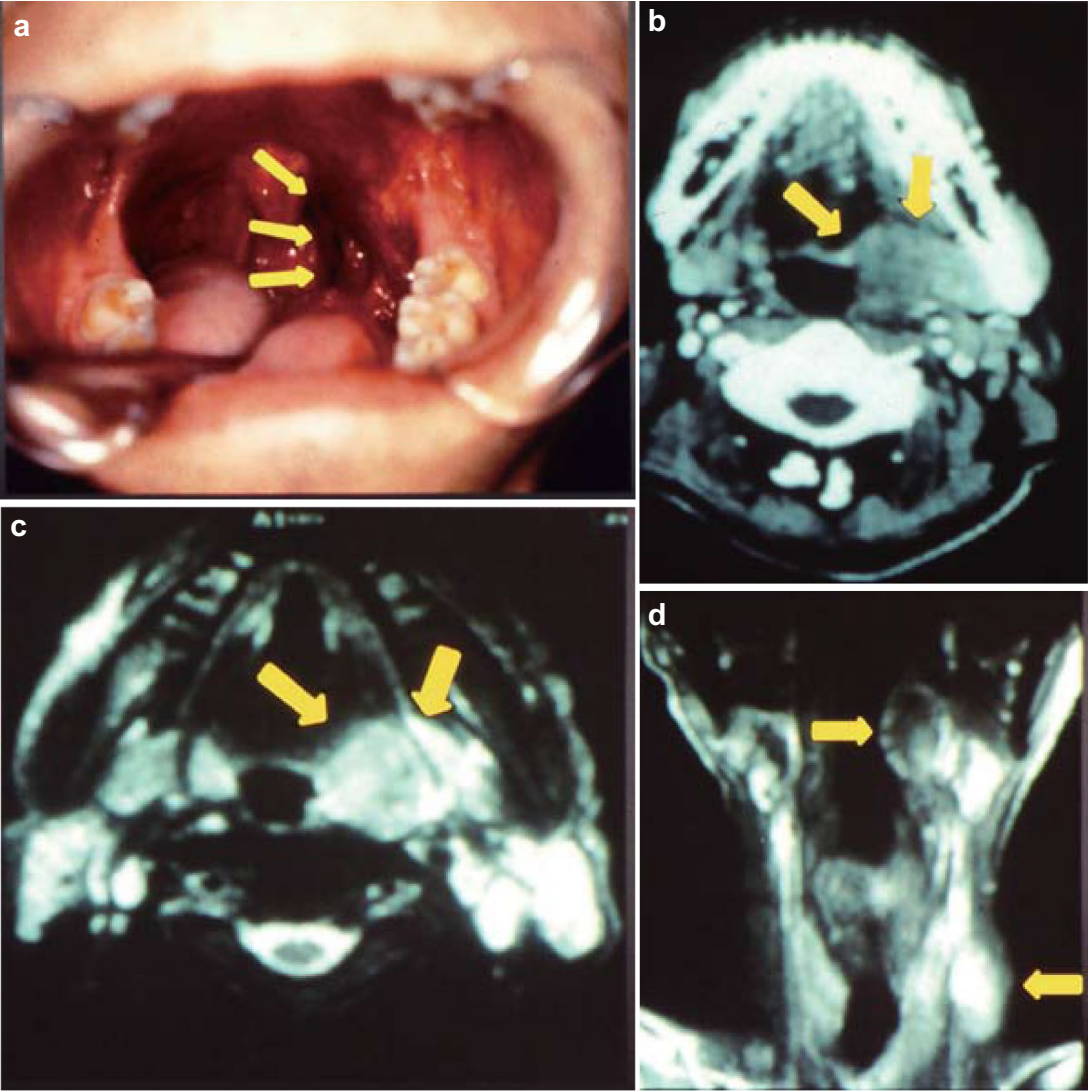
Case 2. **a** Oropharyngeal image. An ulcerated tumor was observed in the left palatine tonsil (*yellow arrow*). An enhanced tumor was observed in the left palatine tonsil on the contrast-axial computed tomography scan of the neck (*yellow arrow*) (**b**), contrast-axial magnetic resonance imaging (MRI) scan (*yellow arrow*) (**c**), and contrast-coronal-MRI scan (*yellow arrow*) (**d**)

A 54-year-old man, with a 30-year history of excessive alcohol consumption and smoking, presented with a 10-month history of throat pain and experiencing a mass in his throat. He had no history of weight loss, dysphagia, or dyspnea. On oropharyngoscopy, a tumorous lesion with ulcerating mucosa was found in the left palatine tonsil (Fig. 2a). A contrast computed tomography scan of the neck also revealed a homogeneously enhanced tumor in the left palatine tonsil (Fig. 2b). MRI examination of the neck revealed a 30 × 20 × 38-mm heterogeneously enhanced tumor of the oropharynx (Fig. 2c) and bilateral cervical lymph node metastasis (Fig. 2d). The patient was diagnosed with T2N2bM0 oropharyngeal cancer. A pathologist analyzed the biopsy sample of the left palatine tonsil and diagnosed the lesion as SCNEC. The patient was treated with sequential chemoradiotherapy. Following 4 induction cycles of cisplatin and etoposide, he underwent radiotherapy for the right palatine tonsil and neck (70 Gy). Patient details are summarized in Table 1.

## Pathology

**Fig. 3:**
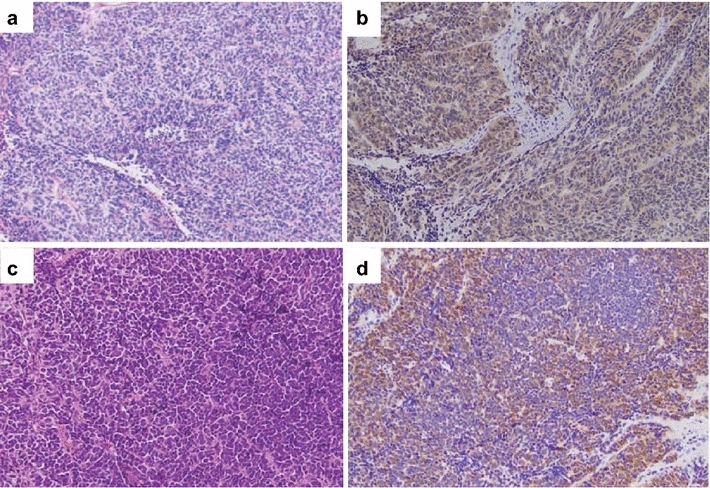
Histologic findings of both patients with small cell carcinoma/neuroendocrine carcinoma of the oropharynx. **a** Hematoxylin and eosin (HE) staining of case 1. Tumor cells had small, round nuclei and scant cytoplasm. **b** Immunohistochemistry (IHC) for p16 in specimens from case 1 shows strong immunoreactivity. **c** HE staining of case 2. **d** IHC staining for p16 in specimens from case 2

Hematoxylin-eosin staining revealed a component with more anaplastic features typical of SCNEC, such as sheets of tightly packed anaplastic cells with round nuclei and scant cytoplasm (Fig. 3a, c). Immunohistochemical analyses revealed that malignant cells expressed high levels of cytoplasmic p16 (Fig. 3b, d). Positivity for p16 was defined by strong and diffuse nuclear and cytoplasmic staining in more than 70 % of cells. Both cases exhibited a high p16-positive/Rb-negative/cyclin D1-negative immunophenotype (Additional file 1: Figure S2). Neuroendocrine differentiation was investigated using immunohistochemical techniques. Both cases showed neuroendocrine features, including staining for synaptophysin, chromogranin A, and CD56 (Table 1). The following primary antibodies were used: p16 (clone G175-405; BioGenex), cyclin D1 (clone SP4; NICHIREI), Rb (clone PDM111; DBP), AE1/AE3 (clone AE1/AE3; Leica), CD3 (clone LN10; Leica), CD56 (clone 1B6; Leica), CD79a (clone JCB117; Dako), synaptophysin (clone 27G12; Leica), and chromogranin A (Dako).

## Transmission electron microscopy studies

**Fig. 4:**
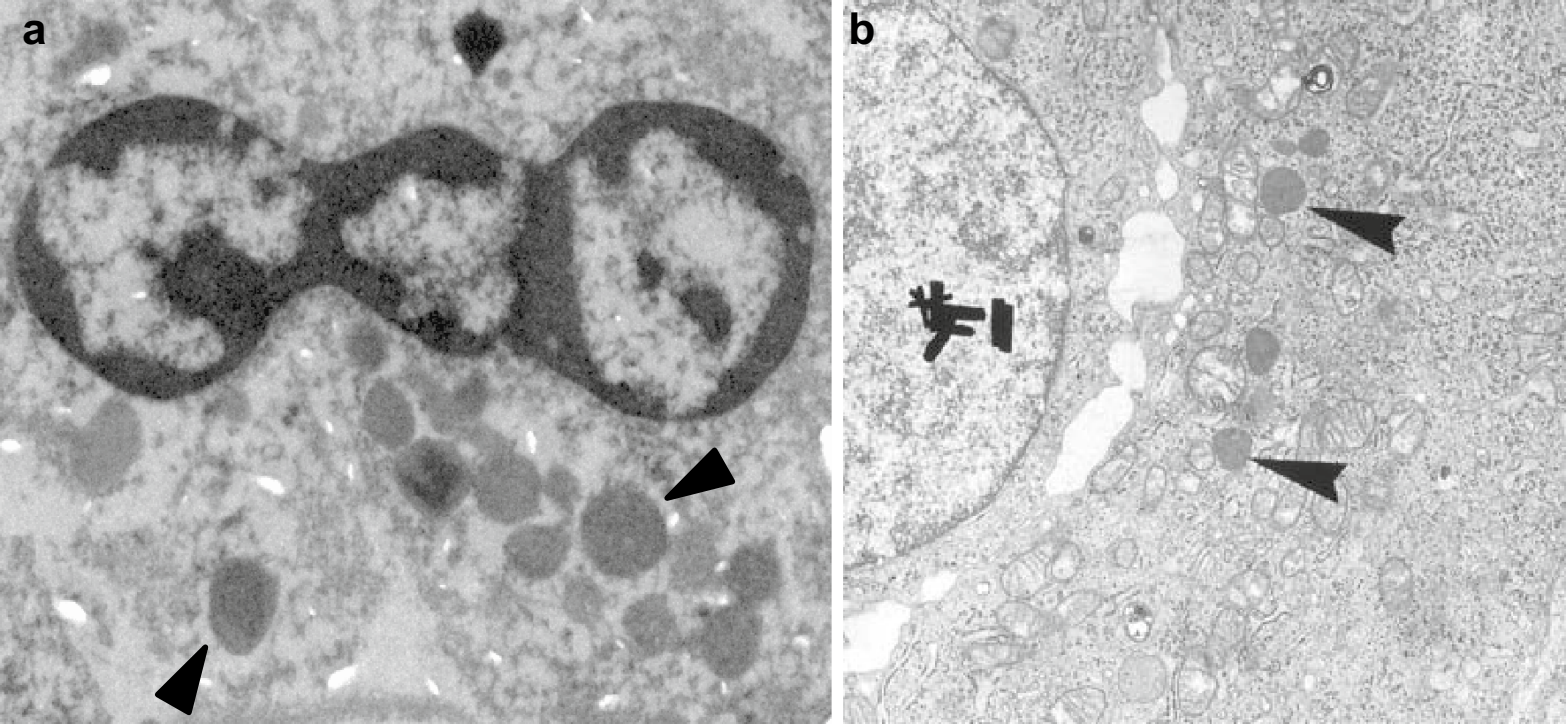
Transmission electron microscopy (TEM) demonstrates dense-core neurosecretory granules (*black arrowheads*). **a** TEM of a specimen from case 1. **b** TEM of a specimen from case 2

Transmission electron microscopy (TEM) studies were performed for both cases to detect neurosecretory granules. TEM specimens were fixed in 2 % phosphate buffered glutaraldehyde overnight, rinsed in 0.1 mol/L phosphate buffer (pH 7.4), post-fixed in 1 % phosphate buffered osmium tetroxide, and dehydrated in epoxy resin. Semi-thin sections from selected areas were stained with uranyl acetate and lead citrate and examined using TEM. TEM evaluation revealed dense-core neurosecretory granules in the cytoplasm of cells in the specimens of both patients (Fig. 4a, b).

## DNA extraction, HPV polymerase chain reaction, and HPV type sequencing

DNA was isolated from the SCNEC elements of specimens obtained during surgery. Genomic DNA was extracted from frozen tumor specimens using the QIAamp DNA Mini Kit (QIAGEN, Hilden, Germany), according to the manufacturer’s instructions.

**Fig. 5:**
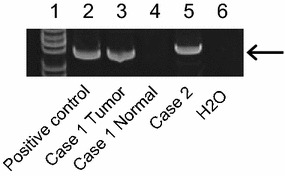
Electrophoresis to assess human papillomaviruses (HPV) status. The bands in *lane 3* represent positive reactivity for HPV-16 in case 1. The band in *lane 4* represents negative reactivity for HPV in adjacent normal mucosal tissues of case 1. The bands in *lane 5* represent positive reactivity for HPV-18 in case 2. *Lane 2* contains an extract from a control cell line that is known to be HPV-16-positive (UMSCC-47; a gift from Dr. TE Carey). The DNA size marker is located in *lane 1*. The water blank is in *lane 6*

HPV status was determined using the HPV Typing Set (Takara Bio., Tokyo, Japan), a primer set for polymerase chain reaction (PCR) specifically designed to identify HPV genotypes 16, 18, 31, 33, 35, 52, and 58 using genomic DNA. The PCR HPV Typing Set method was performed according to the manufacturer’s instructions. Specimens from both patients tested positively for high-risk HPV. After amplification, HPV typing revealed a 238-bp band for HPV-16 in case 1 and a 268-bp for HPV-18 in case 2 (Fig. 5). PCR products were extracted and sequenced using a computed automatic DNA sequencer (ABI PRIZM 3100 Genetic Analyzer; Applied Biosystems, ABI, CA) (Additional file 2: Figure S1).

## Discussion

The worldwide incidence and prevalence of HPV-associated oropharyngeal cancer have been increasing over time. Among the head and neck regions, SCNEC most commonly arises in the larynx, but it has also been reported in the sinonasal tract and salivary glands (Renner 2007; Mineta et al. 2001). SCNECs of the oropharynx are extremely rare, and only 40 cases have been reported since it was first identified by Koss et al. (1972) (Wang et al. 2014; Watson et al. 2013; Kraft et al. 2012; Bishop and Westra 2011). Recently, an association between oropharyngeal SCNEC and high-risk HPV infection was reported (Watson et al. 2013; Kraft et al. 2012; Bishop and Westra 2011). An oncogenic HPV status has been described in 14/19 (73.7 %) cases of oropharyngeal SCNEC (Watson et al. 2013; Kraft et al. 2012; Bishop and Westra 2011). HPV infection confers a better prognosis for patients with oropharyngeal SqCC and basaloid SqCC (Gillison et al. 2000; Jacobi et al. 2015). However, the prognosis of patients with SCNEC of the oropharynx is poor, as the majority of patients die of the disease mainly due to systemic metastasis (Renner 2007; Wang et al. 2014).

Gynecologic extrapulmonary SCNECs most commonly arise in the cervix, and SCNECs of the uterine cervix comprise less than 3 % of cervical cancers (Cohen et al. 2010). SCNECs of the uterine cervix are highly aggressive with extensive local invasion and distant metastases. Most small cell carcinomas of the uterine cervix exhibit neuroendocrine differentiation and ultrastructural examination may be considered the most reliable method for confirming this feature (Ishida et al. 2004). HPV has been detected in more than 90 % of SCNEC tumors through PCR sequencing analysis (Wang and Lu 2004; Horn et al. 2006; Masumoto et al. 2003; Ishida et al. 2004). Many studies have shown that SCNECs that express the HPV oncoproteins E6 and E7 have high levels of p16 (Wang and Lu 2004; Horn et al. 2006; Masumoto et al. 2003). In general, all SCNECs tend to be locally aggressive and have a strong propensity for both regional and distant metastases (Renner 2007).

The diagnosis of SCNEC requires immunohistochemical or ultrastructural studies. With routine hematoxylin and eosin staining, SCNEC exhibits morphology similar to that of nonkeratinizing SqCC. A case with a histopathologic finding of small round cells with scant cytoplasm should be evaluated for synaptophysin, chromogranin A, and CD56 expression, followed by ultrastructural analysis to detect neurosecretory granules in the tumor cells (Mineta et al. 2001). Recently, Alos et al. reported an inverse association between p16 expression and expression of Rb and cyclin D1 in SCNEC. The most prevalent phenotype was high p16-positive/Rb-low or -negative/cyclin D1-low or -negative expression (14/19, 73.7 %). Thus, patchy p16 positivity/strong Rb nuclear staining/strong cyclin D1 nuclear staining was observed in 5/19 cases (26.3 %) (Alos et al. 2016).

Owing to the rarity of these tumors, recommendations for the management of SCNEC of the oropharynx have not been established (Barbeaux et al. 2006). Most patients die within 2 years of diagnosis, despite being treated with adjuvant radiation and chemotherapy (Aggarwal et al. 2010). On the basis of comparative treatments for SCNEC of the larynx and lungs, various modalities have been indicated for patients with SCNEC of the oropharynx (Jaiswal and Hoang 2004).

In conclusion, SCNEC of the oropharynx is extremely rare and highly aggressive, with a poor prognosis. Herein, we present two cases of SCNEC, focusing on its histologic features and treatment modalities. More studies are required to elucidate the pathophysiology of HPV-associated SCNEC in different organ systems.

## Additional files

**Additional file 1: Figure S2.** Expression of Rb and cyclin D1 (A) Absolutely negative Rb expression in case 1. (B) Absolutely negative cyclin D1 expression in case 1. (C) Rb expression staining in case 2. (D) IHC staining for cyclin D1 in specimens from case 2.

**Additional file 2: Figure S1.** (A) Electropherogram of a case 1 extract that contains a relative sequence of the human papillomaviruses (HPV)-16 E7 region. (B) Electropherogram of a case 2 extract that contains a relative sequence of the HPV-18 E7 region.
